# SIRT2 Contributes to the Resistance of Melanoma Cells to the Multikinase Inhibitor Dasatinib

**DOI:** 10.3390/cancers11050673

**Published:** 2019-05-14

**Authors:** Iwona Karwaciak, Anna Sałkowska, Kaja Karaś, Marta Sobalska-Kwapis, Aurelia Walczak-Drzewiecka, Łukasz Pułaski, Dominik Strapagiel, Jarosław Dastych, Marcin Ratajewski

**Affiliations:** 1 Laboratory of Transcriptional Regulation, Institute of Medical Biology, Polish Academy of Sciences, 93-232 Lodz, Poland; isachrajda@cbm.pan.pl (I.K.); lpulaski@uni.lodz.pl (Ł.P.); 2 Laboratory of Epigenetics, Institute of Medical Biology, Polish Academy of Sciences, 93-232 Lodz, Poland; annaswiderek1@wp.pl (A.S.); kaja.karas@gmail.com (K.K.); 3 Biobank Lab, Department of Molecular Biophysics, Faculty of Biology and Environmental Protection, University of Lodz, 90-237 Lodz, Poland; marta.sobalska@biol.uni.lodz.pl (M.S.-K.); dominik.strapagiel@biol.uni.lodz.pl (D.S.); 4 BBMRI.pl Consortium, 54-066 Wroclaw, Poland; 5 Laboratory of Cellular Immunology, Institute of Medical Biology, Polish Academy of Sciences, 93-232 Lodz, Poland; adrzewiecka@cbm.pan.pl (A.W.-D.); jdastych@cbm.pan.pl (J.D.)

**Keywords:** melanoma, SIRT2, sirtuins, tyrosine kinase, resistance

## Abstract

Malignant melanoma is the most aggressive skin cancer and can only be cured if detected early. Unfortunately, later stages of the disease do not guarantee success due to the rapid rate of melanoma cell metastasis and their high resistance to applied therapies. The search for new molecular targets and targeted therapy may represent the future in the development of effective methods for combating this cancer. SIRT2 is a promising target; thus, we downregulated *SIRT2* expression in melanoma cells in vertical growth and metastatic phases and demonstrated that sirtuin acts as regulator of the basic functions of melanoma cells. A detailed transcriptomic analysis showed that SIRT2 regulates the expression of multiple genes encoding the tyrosine kinase pathways that are molecular targets of dasatinib. Indeed, cells with low *SIRT2* expression were more susceptible to dasatinib, as demonstrated by multiple techniques, e.g., neutral red uptake, 3/7 caspase activity, colony formation assay, and in vitro scratch assay. Furthermore, these cells showed an altered phosphorylation profile for proteins playing roles in the response to dasatinib. Thus, our research indicates new, previously unknown SIRT2 functions in the regulation of gene expression, which is of key clinical significance.

## 1. Introduction

Malignant melanoma is the most aggressive skin cancer, and its incidence is increasing each year [1]. Standard chemotherapy with dacarbazine and immunotherapy with recombinant IL-2 or INFα provide low response rates, and have no effect on the overall survival of patients [2,3,4,5,6]. Combination therapy with several compounds used concurrently, e.g., cisplatin, vinblastine, and dacarbazine (CVD regiment) or cisplatin, dacarbazine, carmustine, and tamoxifen (DBCT regiment, or Darmouth regimen), shows a greater response rate percentage [7,8,9], but does not significantly influence overall survival [3]. Failures in the treatment of advanced melanomas are caused by the intrinsic resistance of the cells to known chemotherapeutic agents, which may be related to melanocyte physiology, particularly the production of melanin. This metabolic process generates many highly cytotoxic compounds that create the endogenous melanogenic cytotoxicity (EMC) to which melanocytes are well adapted [10,11]. It is possible that the factor(s) that allows a normal cell to perform its function also supplies a cancer cell with a weapon that we are not yet able to defeat. The exact processes by which resistance is acquired by melanoma cells are, to a great extent, unknown, and our knowledge in this area is greatly limited. There is evidence that the regulation of apoptosis-related processes by overexpression of some proteins, such as ABC transporters, may have significance [12,13,14,15,16]. Other potential mechanisms of resistance include the altered expression of transcription factors (e.g., MITF) [17] and the interaction of melanoma cells with fibroblasts and macrophages in the tumor microenvironment [18,19,20,21].

As mentioned earlier, standard therapy for melanoma patients provides poor results. Nevertheless, understanding the molecular mechanisms by which melanoma cells acquire the ability to undergo uncontrolled proliferation has facilitated the discovery of new therapeutic targets and the development of targeted therapy. A high percentage (66%) of malignant melanomas carry the V600E BRAF gene mutation [22]. This mutation results in constitutive expression of the active protein activating the MAPK pathway via MEK1/2 and ERK1/2 [23] and dysregulation of the control of proliferation, differentiation, adhesion and migration [21,24]. This discovery led to the development of mutant-specific inhibitors of BRAF, vemurafenib (PLX4032) [25,26] and dabrafenib [27], which provided better clinical responses, including improved overall survival of melanoma patients compared with standard chemotherapy regimens [28,29]. Although the use of new inhibitors has improved chemotherapy for melanomas, the clinical response to these novel drugs is not permanent, and typically lasts for 6–8 months due to the acquisition of drug resistance by mutational or altered genetic expression events that reactivate the MAPK pathway [30,31,32,33,34].

Among potential molecular therapeutic targets in melanoma that have recently been gathering attention are sirtuins [35,36], which are NAD(+)-dependent, multifunctional lysine deacylases characterized by a conserved catalytic domain, the sirtuin core domain, and N- and C-terminal domains that differ in different proteins [37,38,39,40]. Different sirtuins exhibit different intracellular localizations. SIRT3, 4 and 5 are localized in mitochondria [41], while SIRT1, 2, 6 and 7 are considered nuclear proteins [42,43]. However, SIRT1 and 2 can also localize in the cytoplasm [44,45]. Most studies of sirtuin functions in melanoma have concentrated on SIRT1 [46,47,48]; however, our group [49] and others [50] have also demonstrated the possible involvement of SIRT2 in the multidrug resistance of melanomas. Using in vitro melanoma cell line-based screening, we previously identified an inhibitor of SIRT2, AC-93253, as a compound that downregulates the expression of genes involved in melanoma progression and chemoresistance acquisition [49]. This compound was subsequently also identified as an SRC inhibitor [51]. These findings prompted us to perform a more detailed analysis of the role of SIRT2 in melanoma cells. We created melanoma cell lines with downregulated *SIRT2* expression in the early and metastatic phases and examined their gene expression and resistance to dasatinib treatment. We found that downregulation of *SIRT2* drastically altered the gene expression profiles of melanoma cells and sensitized them to dasatinib, suggesting a potential role for SIRT2 in the melanoma therapy failures of this drug.

## 2. Results

### 2.1. Identification of SIRT2-Dependent Genetic Information in Melanoma Cells

**Figure 1:**
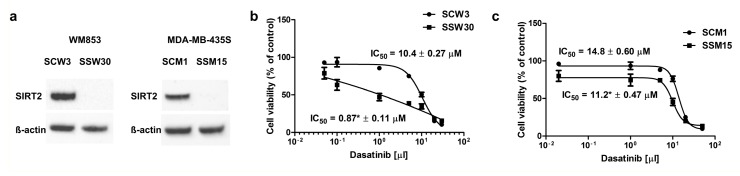
Cellular WM853 and MDA-MB-435S clone characteristics. (**a**) SIRT2 expression in SCW3 and SSW30 clones of WM853 cells and SCM1 and SSM15 clones of MDA-MB-435S as evidenced by Western blotting. (**b**) The cytotoxic effects of dasatinib treatment (48 h) on melanoma SCW3 and SSW30 clones of the WM853 cell line, as evidenced using the neutral red assay, mean ± SD, (*n* = 3, independent experiments) (**c**) The cytotoxic effects of dasatinib treatment (48 h) on melanoma SCM1 and SSM15 clones of the MDA-MB-435S cell line, as evidenced using the neutral red assay, mean ± SD, (*n* = 3, independent experiments).

To characterize the role of *SIRT2* in melanoma cells, we downregulated the expression of this gene in two human melanoma cell lines: WM853 and MDA-MB-435S. Because earlier work has suggested that the role of SIRT2 may vary depending on the stage of disease [52], we chose two cell lines representing different stages of disease development (primary/vertical growth P/VG—WM853 and metastasis—MDA-MB-435S). When SIRT2 expression was analyzed at the protein level, the protein was below the detection level in clones transfected with SIRT2 shRNA. However, SIRT2 protein levels were the same in maternal lines in cells transfected with the control shRNA (Figure 1a). Initially, inhibition of *SIRT2* expression significantly affected the phenotype in both cell lines (Figure S1a). This finding encouraged us to analyze cellular clones for gene expression profiles. To achieve this goal, RNA-seq analysis of control cells and cells with downregulated *SIRT2* expression was performed. The resultant data were processed with three bioinformatics software packages: DESeq v1.32 [53], DESeq2 v1.20.0 [54] and edgeR v3.1 [55]. To identify the most significant hits, we considered only genes that were identified in all three bioinformatics analyses (to show how each tool overlaps with other tools, Venn diagrams were used, Figure S2). In WM853 and MDA-MB-435S cells, we found 3550 and 624 differentially expressed transcripts, respectively. Gene ontology analysis revealed that GO terms related to adhesion, migration, differentiation, and proliferation were overrepresented (in both melanoma cell lines) among the differentially expressed genes (Table S1 and Dataset S3). Comparisons of changes in gene expression resulting from *SIRT2* depletion in the two examined cell lines showed that the expression of certain genes was altered in the same direction in the considered cell lines (e.g., *ITGA1*, *EGFR*, *EPHA2*, and *CDKN2D* were downregulated, and *FGFR1* was upregulated in both cell lines), while the expression of other genes showed an opposite direction of change (e.g., *SDC2* and *NCAM1*, upregulated in WM863 and downregulated in MDA-MB-435S; *AXL*, downregulated in WM863 and upregulated in MDA-MB-435S). They also included genes with altered expression in only one of the *SIRT2*-depleted cell lines (Table 1 and Table 2). A list of the genes that were differentially expressed included several genes coding adhesion receptors associated with cell migration, proliferation, and metastasis (integrins, cadherins, selectins, syndecans) [56,57,58,59] and cell cycle regulators [60,61,62] (Table 1 and Table 2). A particularly interesting category of differentially expressed genes comprised those encoding receptor tyrosine kinases as well as other signaling proteins involved in the MAP/ERK pathway, indicating that this particular signaling pathway might be directly or indirectly controlled by SIRT2 (Table 1 and Table 2). To assess whether the genes identified in the RNA-seq analysis were subject to expression changes in other melanoma cells when *SIRT2* was inhibited, we treated A375 cells (stage: metastasis) with the SIRT2 inhibitor thiomyristoyl [63]. In contrast to other SIRT2 inhibitors, this compound inhibits deacetylation and demyristoylation functions of SIRT2 [64]. The pharmacological inhibition of SIRT2 in A375 resulted in significant inhibition of *EGFR*, *EPHA2*, *ITGA1*, *SDC2*, *DDR1* and *EPHB1* that was similar to the SS15 clone in MDA-MB-435S cells (Table S2).

### 2.2. SIRT2-Inhibition Sensitizes Melanoma Cells to Dasatinib

Analyses of the transcriptomes of the created *SIRT2*-deficient cell lines revealed the altered expression of numerous genes identified as targets of dasatinib (e.g., *EGFR*, *EPHA2*, *EPHB1*, *BTK*, *MAP3K4*, *MAP3K14*, *DDR1*, *GAK*, and *LYN*) [65,66] (Table 1 and Table 2), which prompted us to examine whether *SIRT2* downregulation could sensitize melanoma cells to dasatinib. Cells with low expression of *SIRT2* were more sensitive to dasatinib treatment compared with those with normal expression of the gene (Figure 1b,c), and this effect was accompanied with higher susceptibility to early membrane damage and apoptosis (Figure S3). However, it should be noted that the effects of dasatinib were much stronger in the P/VG cell line than in the cell line representing the metastasis stage.

**Figure 2:**
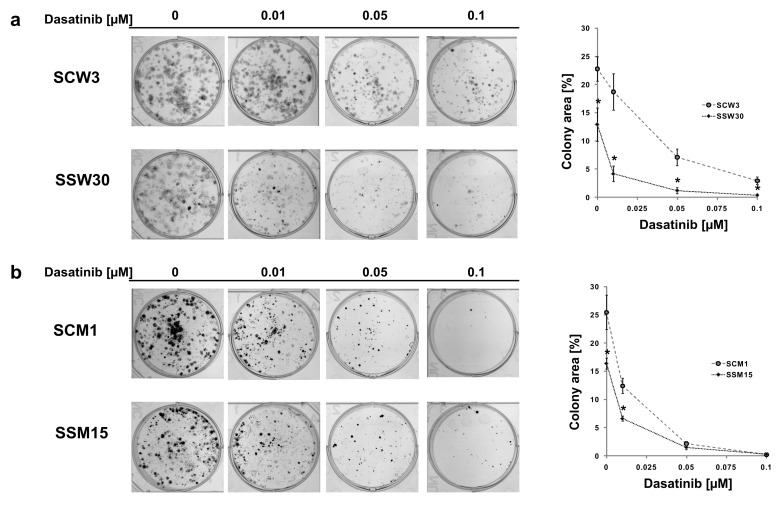
(**a**) Effect of dasatinib on the capacity of SCW3 and SSW30 cells to produce progeny as determined using the colony formation assay. Images from a single representative experiment (left panel) and a computer-generated area lacking values to illustrate the colony formation assay (right panel), mean ± SD, (*n* = 4). * Indicates a statistically significant difference at *p* < 0.05. (**b**) Effect of dasatinib on the capacity of SCM1 and SSM15 cells to produce progeny as determined using the colony formation assay. Image from a single representative experiment (left panel) and computer-generated area lacking values to illustrate the colony formation assay (right panel), mean ± SD, (*n* = 4). * Indicates a statistically significant difference at *p* < 0.05.

**Figure 3:**
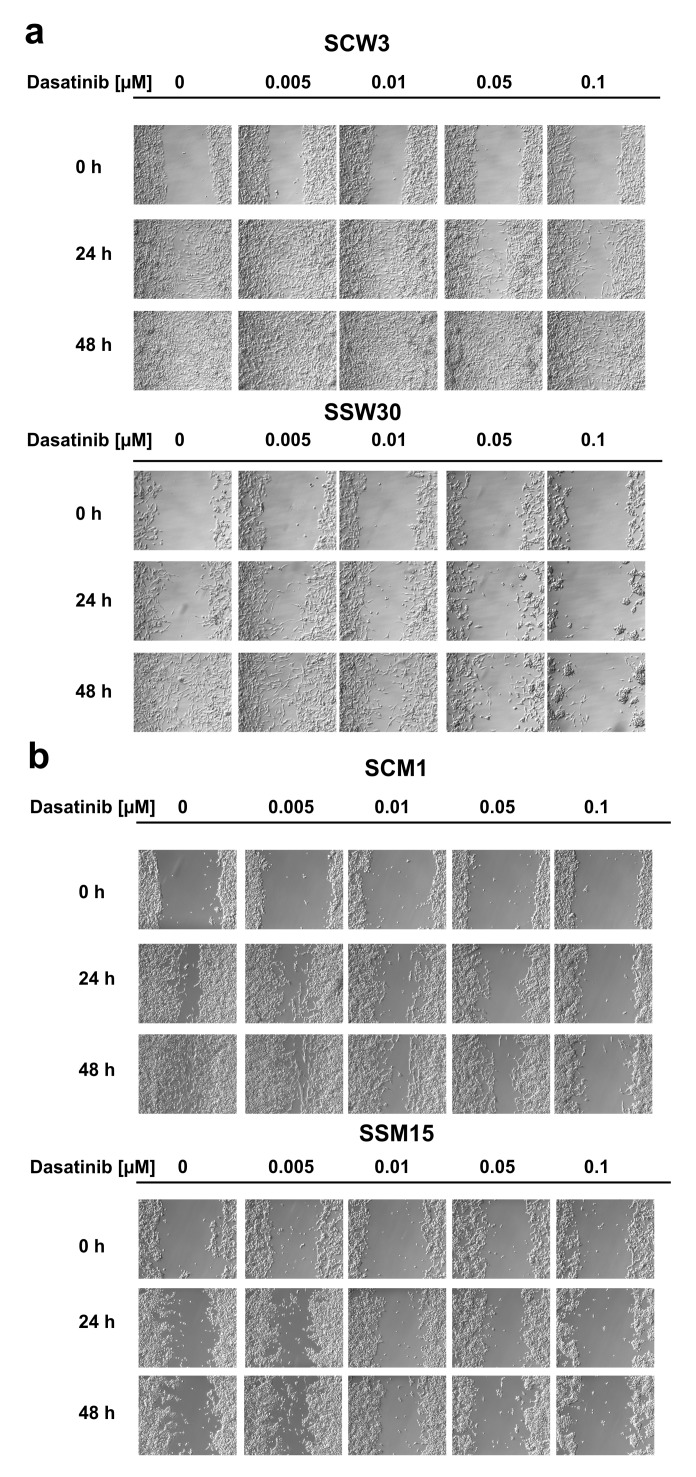
Treatment with dasatinib impairs the migration of melanoma cells based on the scratch assay. (**a**) Image from a single representative experiment performed using WM853 SCW3 and SSW30 clones. (**b**) Image from a single representative experiment performed using MDA-MB-435S SCM1 and SSM15 clones. Quantification of above results is shown in the Figure S4. 10 × 10 magnification.

To confirm the differences in the reproductive viability of cells with low *SIRT2* expression following dasatinib treatment, we performed colony formation assays. Inhibition of *SIRT2* expression had a much greater impact on the ability to form colonies in the P/VG line (WM853), and treatment with dasatinib further reduced the cell potential for colony formation (Figure 2a). In the metastatic melanoma line, inhibition of *SIRT2* expression had a slightly reduced effect on colony formation in the control-treated cells, and treatment with dasatinib had much smaller effect on colony formation in the *SIRT2*-deficient cell line (Figure 2b) than in the P/VG line. We next investigated the effects of the combination of *SIRT2* knockout and dasatinib treatment on the migratory function of melanoma cells using the scratch assay. Our results showed that lack of *SIRT2* expression in the P/VG melanoma line significantly inhibited cell motility by 4.5-fold (24 h) to 470-fold (48 h), and treatment with dasatinib strongly increased the observed inhibitory effects of *SIRT2* depletion (Figure 3a and Figure S4a). In metastatic cells (Figure 3b and Figure S4b), the lack of *SIRT2* expression caused 2.5-fold and 5-fold inhibition of migration, but the effect of dasatinib in *SIRT2*-deficient cells seems to be reduced compared to that of control cells. One possible explanation is that the lack of *SIRT2* decreased the migratory potential of cells to the extent where any additional inhibitory effects are difficult to observe.

### 2.3. Effects of SIRT2 Inhibition and Dasatinib on the Cell Cycle Distribution

**Figure 4:**
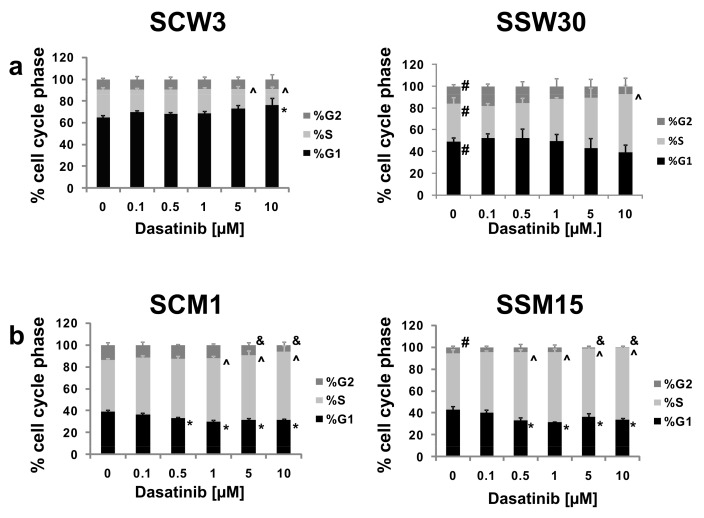
(**a**) Cell cycle phase distribution in SCW3 and SSW30 clones. (**b**) Cell cycle phase distribution in SCM1 and SSM15 clones. The data are presented as the mean ± SD, (*n* = 3). * Indicates a statistically significant difference at *p* < 0.05 in %G1 phase between dasatinib and control treatment. ^ Indicates a statistically significant difference in %S phase between dasatinib and control treatment at *p* < 0.05. & Indicates a statistically significant difference at *p* < 0.05 in %G2 phase between dasatinib and control treatment. # Indicates a statistically significant difference at *p* < 0.05 for a given phase between control cell line and SIRT2-deficient cells.

To determine the effects of *SIRT2* knockdown and dasatinib treatment on the cell cycle, quantification of the percentage of cells in different cell cycle phases was determined after 24 h of dasatinib treatment. In control WM853 SCW3 cells, 10 μM dasatinib treatment caused an increase in the percentage of cells in G1 phase and a decrease in the percentages of cells in S phase (for 5 and 10 μM) (Figure 4a). When analyzing the cell cycle distribution in the WM853 SSW30 clones, we noticed that these cells showed a reduced percentage of cells in G1 phase and an increased percentage of cells in S and G2 phases compared to control SCW3 cells. Treatment with dasatinib (especially at high concentrations) led to an increase in the percentage of cells in S phase (10 μM of tested compound) (Figure 4a). For metastatic MDA-MB-435S SCM1 cells, dasatinib decreased the percentage of cells in G1 (0.5–10 μM dasatinib) and G2 phases (5–10 μM dasatinib) and increased those in S phase (1–10 μM dasatinib). MDA-MB435S SSM15 cells showed a reduction in G2 phase cells. Treatment of these cells with dasatinib led to decreases in the percentage of cells in G1 and G2 (0.5–10 μM dasatinib) phases (up to almost complete elimination of cells in G2 phase) and increases in the percentage of cells in S phase (5–10 μM dasatinib) (Figure 4b).

### 2.4. Modulation of Signaling Pathways by SIRT2 and Dasatinib

**Figure 5:**
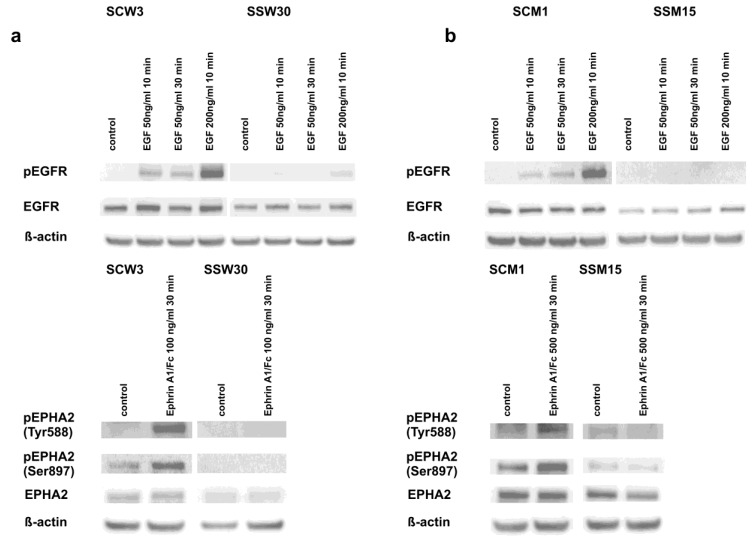
*SIRT2* downregulation impairs the response of melanoma cells to EGFR and EPHA2 activators. The generated melanoma clones were treated with selected concentrations of EGF (EGFR activator) and ephrin A1 (EPHA2 activator) for 10 min and/or 30 min, respectively. Then, protein lysates were prepared and analyzed by Western blotting. (**a**) Experiments performed using WM853 SCW3 and SSW30 clones. (**b**) Experiments performed using MDA-MB-435S SCM1 and SSM15 clones.

**Figure 6:**
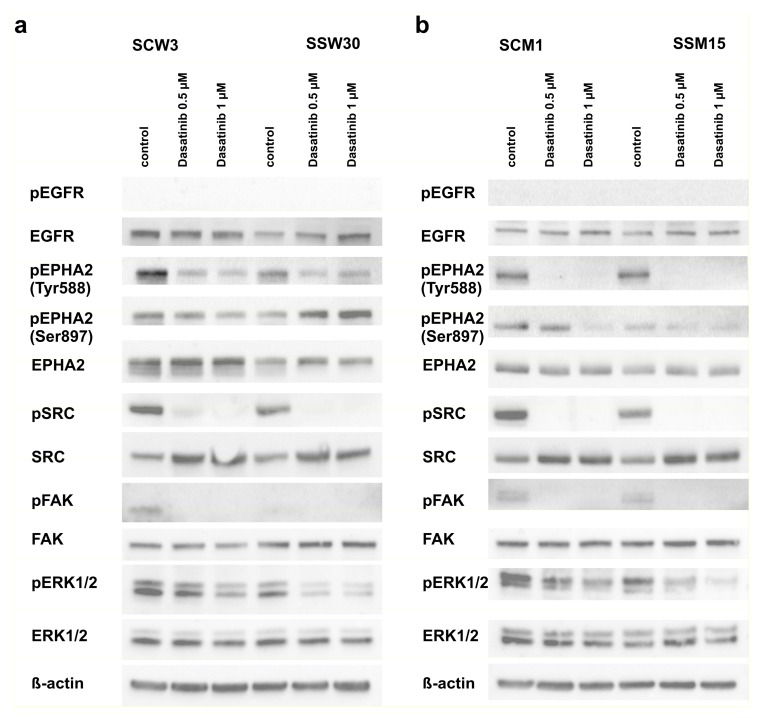
*SIRT2* downregulation impairs the expression and phosphorylation of tyrosine kinase receptor-associated pathways. The generated melanoma clones were treated with selected concentrations of dasatinib for 1 h. Then, protein lysates were prepared and analyzed by Western blotting. (**b**) Experiments performed using WM853 SCW3 and SSW30 clones. (**a**) Experiments performed using MDA-MB-435S SCM1 and SSM15 clones.

Given the significant role of tyrosine kinase receptors [67,68], downstream signaling of the SRC-ERK [23] pathway in the development of melanomas and their resistance to anticancer drugs and the RNA-seq sequencing results supporting the participation of SIRT2 in the regulation of these pathways, we confirmed that EGFR and EPHA2 receptors were downregulated in cells with low *SIRT2* expression (Figure 5a,b and Figure 6a,b and Figure S1b). Although we did not observe the intrinsic phosphorylation of EGFR in any cell line, the differences in the response of the melanoma cells with *SIRT2*-downregulation to EGF was evident (Figure 5a,b). Similarly, the lack of response of *SIRT2*-deficient cells to Ephrin-A1 indicates that signal transduction through the EPHA2 receptor in these cells is impaired. Furthermore, we decided to examine the mechanism by which *SIRT2* downregulation and dasatinib affects the phosphorylation of downstream elements of the EGFR and EPHA2 pathways. The intrinsic phosphorylation of EPHA2 on Tyr588 decreased in SSW30 clones, and treatment with dasatinib further decreased the phosphorylation. However, a different pattern was observed in analyses of Ser897 phosphorylation. In SSW30 cells, slightly higher phosphorylation was detected that increased by dasatinib. In control SCW3 cells, dasatinib led to reduced phosphorylation on Ser897 (Figure 6a). In contrast, in both MDA-MB-435S clones, there were no differences in the intrinsic phosphorylation of EPHA2 on Tyr588, but dasatinib led to phosphorylation inhibition (Figure 6b). SSM15 clones had a lower phosphorylation ratio on Ser897 of EPHA2, and in agreement with our assumptions, the inhibitory effect of dasatinib was stronger in these clones than in the control SCM1 clones (Figure 6b). Next, we analyzed the phosphorylation of the downstream kinases SRC and ERK1/2. We observed that in cells with normal *SIRT2* expression, the intrinsic phosphorylation of SRC and ERK1/2 was higher than in cells with *SIRT2* knockdown. Treatment with dasatinib increased this effect. We also analyzed the phosphorylation of focal adhesion kinase (FAK), which is crucial for the induction of apoptosis and inhibition of the migratory functions of melanoma cells [69,70]. In both cell lines with low *SIRT2* expression, we observed decreased phosphorylation of FAK kinase (Figure 6a,b) that was further decreased by treatment with dasatinib.

## 3. Discussion

Failures in the treatment of advanced melanoma require new approaches and a search for new therapeutic targets that may increase the effectiveness of therapy, similar to the discovery of the V600E BRAF mutant [25]. NAD(+)-dependent, multifunctional lysine deacylases sirtuins are among such potential molecular targets [36]. Our previous studies using the SIRT2 inhibitor have shown that SIRT2 might be involved in intracellular processes that mediate melanoma proliferation and multidrug resistance [49], prompting us to further investigate the role of this deacetylase in the biology of melanoma cells. Thus, we created melanoma cell lines with silenced *SIRT2* expression. We chose the WM853 line, representing the primary/vertical growth stage and MDA-MD-435S representing metastatic melanoma as previous studies indicated that the role of SIRT2 might change with the advancement of cancer development [52]. Transcriptome analysis demonstrated that SIRT2 has important regulatory roles in the expression of multiple genes associated with the molecular signatures of melanoma progression [71]. Among the genes that were downregulated by silencing this deacetylase are those that have been positively correlated with melanoma aggressiveness [71] (e.g., *NCAPG*, *CDKN2C*, *GINS1*, and *DHFR* are all downregulated in the SSW30 clone, Dataset S1). Among genes that were upregulated by *SIRT2* silencing were those that are downregulated in aggressive melanoma cells (e.g., *MGST1* is upregulated in the SSW30 and SSM15 clones, Datasets S1 and S2). Similarly, genes related to melanoma invasion were downregulated in *SIRT2*-deficient melanoma cell lines (e.g., *HLA-DRA*, *IL-6*, *CD74*, and *HLA-DRB1* in the SSW30 clone, Dataset S1; *PTPRZ1*, *FST*, and *NRCAM* in the SSM15 clone, Dataset S2). Interestingly, although the two cell lines represented different stages of disease, we obtained similar levels of *SIRT2* silencing. In WM853 cells, substantially more genes were differentially regulated than in metastatic MDA-MB-435S cells. This finding might indicate that in the VG phase, SIRT2 plays a more prominent regulatory function associated with the acquisition of metastatic properties and suggests that SIRT2 in tumors in this phase could represent a better therapeutic target compared with those in the metastatic phase. Among the differentially expressed genes, we found numerous genes that are molecular targets for dasatinib [65]. Consistent with this observation, both melanoma cell lines with silenced *SIRT2* expression showed greater susceptibility to dasatinib than control cells as evidenced by several experimental approaches, including neutral red uptake, caspase 3/7 activity, and clonogenic assays (Figure 1, Figure 2 and Figure S3). We also showed that *SIRT2* loss substantially decreased the migration of melanoma cells, and treatment with dasatinib further increased this inhibition of migration (Figure 3). In analyses of cell percentages in different cell cycle phases, we noticed that inhibition of *SIRT2* expression arrested cells in S phase and that dasatinib enhanced this effect (Figure 4). Taken together, these observations are consistent with the hypothesis that SIRT2 regulates some putative elements necessary for activation of the MAPK pathway in the absence of V600E mutated BRAF-mediated activation. This hypothesis is consistent with RNA-seq results showing that SIRT2 regulates the expression of genes coding for tyrosine kinase receptors and MAPK/ERK pathway signaling molecules (Table 1 and Table 2; Datasets S1 and S2). This hypothesis is also consistent with the observations that dasatinib mediates different protein phosphorylation profiles in *SIRT2*-deficient melanoma cells than in control cells (Figure 5 and Figure 6). Our results are consistent with those of Wilking-Busch et al. [72], who showed that inhibition of *SIRT2* expression in the melanoma A375 cell line led to a decrease in cell proliferation and the ability to form colonies, and they contradict the results of Bajpe et al. [50], who showed that inhibition of *SIRT2* expression was associated with increased ERK phosphorylation and conferred resistance to cetuximab, AZD6244, and vemurafenib. Previous studies have suggested that SIRT2 may act as both a tumor promoter and suppressor [52], [73]. Our previous study [49], the work of Wilking-Busch et al. [72] and this report demonstrate that SIRT2 in melanomas acts as a tumor promoter at least in the context of the tested drugs and is a promising candidate for new anti-melanoma targeted therapies.

Dasatinib was first identified as a Src/Abl kinase inhibitor [74], and subsequent studies have shown that it also targets other kinases [65,75]. Dasatinib exhibits activity against melanomas in vitro [76], but its clinical use in melanoma monotherapy has been disappointing [77]. The use of dasatinib in combination with dacarbazine has provided slightly better results [78], but they remain far from expectations. Our study provides evidence that SIRT2 is one factor responsible for the resistance of melanoma cells to dasatinib. A possible explanation for SIRT2-mediated regulation of melanoma cell sensitivity to dasatinib is the role of this deacetylase in the regulation of genes encoding tyrosine kinase receptors and other signaling molecules that are responsible for the function of the main signaling pathway targeted by this drug [65,75].

In 50% of patients undergoing treatment with vemurafenib [26] or dabrafenib [27] (V600E BRAF inhibitors), resistance to these drugs develops after 6–8 months, which might be due to reactivation of the MAPK pathway [30,31,32,33,34]. Therefore, combination therapy with BRAF inhibitor and trametinib (MEK1/2 inhibitor) has improved clinical outcomes [79], but has not solved the problem of emerging resistance [80]. Activation of survival signaling through receptor tyrosine kinases (RTKs), e.g., EGFR [81,82], is also involved in the observed acquired resistance to the mentioned drugs. Our transcriptomic analysis showed that loss of *SIRT2* in both P/VG and metastatic melanoma cell lines was associated with decreased expression of *EGFR* and *EPHA2*, receptors that are important for the development, proliferation and acquisition of multidrug resistance by melanoma cells [68,81,83,84,85,86,87,88,89,90]. Western blotting confirmed that EPHA2 and EGFR expression was reduced in cells without *SIRT2* and that dasatinib treatment inhibited the phosphorylation of these receptors and the downstream signaling elements Src, ERK1/2 and FAK.

The practical consequences of the hypothesis that SIRT2 regulates genes encoding tyrosine kinase receptors and other signaling molecules that are critical for the function of the main signaling pathway targeted by dasatinib and V600E inhibitors [65,75] should be considered in light of new combination therapies for the treatment of melanoma. One combination worth exploring would be the use of specific inhibitors of SIRT2 together with dasatinib to increase the clinical potential of the latter. Another potential combination would be the SIRT2 inhibitor combined with the vemurafenib-trametinib regimen to increase the time to appearance of resistance to vemurafenib or to completely eliminate this resistance. Unfortunately, to our knowledge, there are no known SIRT2 or other SIRT inhibitors undergoing clinical evaluation for the treatment of cancer to date. The SIRT1 inhibitor EX-527 was tested in a phase II clinical study in Huntington’s disease patients and was well tolerated [91]. The major problem with known SIRT inhibitors is their selectivity [92]. However, the identification of the novel, SIRT2-specific inhibitor SirReal2 with 1000-fold selectivity over SIRTs 1, 3, 4, 5, and 6 [93] is a promising development that may potentially open new avenues for future clinical applications.

## 4. Materials and Methods

### 4.1. Cell Lines, Cell Culture and Reagents

The human melanoma cell lines MDA-MB-435S (stage: metastatic) and A375 (stage: metastatic) were obtained from ATCC (Manassas, VA., USA) and maintained in Dulbecco’s Modified Eagle’s Medium supplemented with 10% fetal bovine serum (PAN-Biotech GmbH, Aidenbach, Germany) at 37 °C in a humidified atmosphere containing 5% CO_2_. The human melanoma cell line WM853 (stage: primary/vertical growth) was purchased from Rockland (Rockland, Limerick, PA, USA) and was maintained in Tumor Specialized Medium containing 80% MCDB153 (Sigma-Aldrich, St. Louis, MO, USA), 20% Leibovitz’s (Sigma-Aldrich, St. Louis, MO, USA), 2% fetal bovine serum (Pan Biotech GmbH, Aidenbach, Germany), 1.68 mM calcium chloride, and 1.2 g (11.2 mM) sodium bicarbonate at 37 °C in a humidified atmosphere containing 5% CO_2_. Human EGF and Ephrin-A1/Fc Chimera were purchased from Sigma-Aldrich. Thiomyristoyl was purchased from Cayman Chemical (Ann Arbor, MI, USA).

### 4.2. Stable SIRT2 Knockdown Melanoma Cell Line Generation

To establish stable SIRT2-silenced cell lines, MDA-MB-435S and WM853 cells were transfected with short hairpin RNA (shRNA) plasmids: scrambled negative control noneffective shRNA (TR30012) or SIRT2 (T301692D) (Origene Tech. Rockville, MD, USA). Transfection was performed with Metafectene PRO (Biontex, München, Germany) following the manufacturer’s instructions. The cells were then cultured by replacing the medium every three days with complete medium containing 1 µg/mL (for MDA-MB-435S) and 0.4 µg/mL (for WM853) puromycin for one month during which the following stable clones were selected: WM853 SCW3, control; WM853 SSW30, downregulated SIRT2; MDA-MB-435S SCM1, control; MDA-MB-435S SSM15, downregulated SIRT2. SIRT2 expression in selected clones was determined by immunoblotting analysis.

### 4.3. RNA Extraction, Reverse Transcription and Quantitative PCR

Cells were lysed using TRI reagent (Sigma Aldrich, St. Louis, MO, USA). Briefly, 5 μg of total RNA was reverse transcribed using the Maxima First Strand cDNA Synthesis Kit for RT-quantitative PCR (Thermo Fisher Scientific, Waltham, MA, USA). Real-time quantitative PCR was performed using SYBR Green I Master Mix (Basel, Switzerland). The reactions were run on a LightCycler 480 from Roche under the following conditions: 95 °C for 5 min followed by 40 cycles of 95 °C for 10 s, 60 °C for 10 s, and 72 °C for 20 s. The following primers were used: *SIRT2*, 5′-GAAGGACAAGGGGCTACTCC-3′ (forward) and 5′-GATATCAGGCTTCACCAGGC-3′ (reverse); *EGFR*, 5′-GTGCAGGAGAGGAGAACTGC-3′ (forward) and 5′-GGCACCAAAGCTGTATTTGC-3′ (reverse); *EPHA2*, 5′-TACGAGAAGGTGGAGGATGC-3′ (forward) and 5′-ACGGCTGTGAGGTAGTGTGG-3′ (reverse); *ITGA1*, 5′-TGGGTGCTTATTGGTTCTCC-3′ (forward) and 5′-AAATCCTCCATTTGGGTTGG-3′ (reverse); *SDC2*, 5′-CTGCTCCAAAAGTGGAAACC-3′ (forward) and 5′-CAGCAATGACAGCTGCTAGG-3′ (reverse); *GAK*, 5′-TCACCAACCTCAAGGACACC-3′ (forward) and 5′-CTTGGAGTCCAGGAACAACC-3′ (reverse); *DDR1*, 5′-GGTCAGGAGGTGATCTCAGG-3′ (forward) and 5′-TGGAGTCGTTGAGGTACACG-3′ (reverse); and *EPHB1*, 5′-CTTTGACCCTCCAGAAGTGG-3′ (forward) and 5′-CTCCACATTGTCGTCACAGC-3′ (reverse). The mRNA levels were normalized to the RPL13A housekeeping gene as described by Vandensompele et al. [94].

### 4.4. Western Blotting

Whole cell lysates were prepared using ice-cold RIPA buffer (50 mM Tris-HCl pH 8.0, 150 mM NaCl, 0.1% Triton X-100, 0.1% SDS, 0.5% sodium deoxycholate) supplemented with Halt Protease Inhibitor Cocktail (Thermo Fisher Scientific). The samples were centrifuged for 15 min at 10,000× *g*. The protein concentration in the supernatant was determined using the Pierce BCA Protein Assay kit (Thermo Fisher Scientific). Total protein was separated on a 12% Bis–Tris NuPage precast gel (Thermo Fisher Scientific) and transferred to a Hybond-C membrane (GE Healthcare Life Sciences, Marlborough, MA, USA) using the iBlot dry blotting system (Thermo Fisher Scientific). The membranes were blocked with 5% milk and incubated overnight with the appropriate primary antibodies at 4 °C and subsequently with HRP-conjugated secondary antibody (ab6721, Abcam, Cambridge, UK) for 1 h at RT. The following primary antibodies were used: anti-SIRT2 (EPR1667) (Abcam), anti-beta Actin (Abcam), EphA2 (phospho Tyr588) (GeneTex, Irvine, CA, USA), Phospho-EphA2 (Ser897) (D9A1) (Cell Signaling, Danvers, MA, USA), EphA2 (D4A2) (Cell Signaling), EGF Receptor (Cell Signaling), Phospho-EGF Receptor (Tyr1068) (D7A5) (Cell Signaling), Src (Cell Signaling), Phospho-Src Family (Tyr416) (Cell Signaling), ERK 1/2 Antibody (C-9) (Santa Cruz, Dallas, TX, USA), and Phospho-ERK (E-4) (Santa Cruz). Specific bands were visualized using SuperSignal West Pico Chemiluminescent Substrate (Thermo Fisher Scientific) and the G-Box chemiluminescence imaging station (Syngene, Cambridge, UK). The signal strength in cognate bands corresponding to the assayed proteins was quantified with G-Box station software. Original scans are available in Supplementary Materials (Original scans of Figure 1, Figure 5 and Figure 6).

### 4.5. Preparation of Libraries, Sequencing and RNA-seq Data Analysis

Global analysis of gene expression in WM853 SCW3, WM853 SSW30, MDA-MBA-435S SCM1 and MDA-MB-435S SSM15 clones was analyzed by high-resolution RNA sequencing (RNA-seq). The procedure applied for the preparation of libraries and sequencing has been described in detail in our previous study [95]. A Venn diagram was used to compare the identified DEGs from the different expression analysis tools and to designate the shared DEGs. An FC cut off value of 1.5-fold was used for the detection of DEGs. RNA-seq results are available under SRA accession: PRJNA530710: https://www.ncbi.nlm.nih.gov/sra/PRJNA530710.

### 4.6. Gene Ontology Analysis

Gene ontology was performed using PANTHER software [96]. The following parameters were used in the analysis: displaying only results for FDR *p* < 0.05; analysis type: PANTHER overrepresentation test; reference list: Homo sapiens (all genes in database); test type: Fisher’s exact; and correction: false discovery rate.

### 4.7. Cell Cycle Analysis

Cells were seeded in 6-well plates. After 24 h, cells were treated with dasatinib for an additional 24 h. Then, the cells were harvested, collected, washed twice in ice-cold PBS and fixed in ice-cold 70% ethanol overnight at 4 °C. The cells were then washed twice in ice-cold PBS, and DNA was stained with a solution containing 10 μg/mL propidium iodide, 0.1% Triton X-100 and 100 μg/mL RNase A at RT for 30 min. The cell samples were measured with a LSRFortessa (Becton–Dickinson, Franklin Lakes, USA) flow cytometer. Data acquisition and analysis were performed using FlowJo 7.6.5 software (Treestar Data Analysis Software., Ashland, OR., USA).

### 4.8. In Vitro Scratch Assay

The migratory potential of the cell lines was assayed using the in vitro scratch assay [97]. Cells were grown on 12-well plates until monolayer formation; then, a scratch was created using a 200-μL pipette tip. The cells were then treated with increasing concentrations of dasatinib. After 24 h and 48 h of incubation, the plates were placed under an Eclipse TE2000 inverted microscope (Nikon, Tokyo, Japan), and images of the scratch were acquired. The data were analyzed using ImageJ (http://imagej.nih.gov/ij/).

### 4.9. Detection of Early Cellular Membrane Damage

Cells were seeded in 96-well plates at a density of 1 × 10^4^ per well. After 24 h, the cells were treated with dasatinib. Staining solution (0.35 μM YO-PRO-1 in an appropriate cell culture medium) prewarmed to 37 °C was added 24 h after the treatment, and the cells were incubated for 30 min. The medium was then removed, and the cells were washed twice with PBS. Cells were fixed with fixation solution (3.7% formaldehyde in PBS) and incubated with nuclear staining solution (10 µg/mL Hoechst 33,342 in PBS) for 20 min. After 3 washes with PBS, the plates were scanned in the ArrayScan VTi HCS Reader (Thermo Fisher Scientific), and the fluorescence of YO-PRO-1 (a marker of cell membrane damage) was analyzed.

### 4.10. Cell Viability Measurements

For the determination of cell viability, the neutral red cytotoxicity assay was used [98], as described in our previous study [99].

### 4.11. Caspase 3/7 Activity Measurements

For the determination of apoptosis, caspase 3 and 7 activities were measured using the Caspase-Glo^®^ 3/7 Assay System from Promega (Madison, WI, USA) according to the manufacturer’s protocol. The luminescence measurements were performed on an Infinite 200 PRO (Tecan, Männedorf, Switzerland) plate reader.

### 4.12. Colony Formation Assay

Cells were seeded in a 6-well plate at a density 1000 cells per well. After incubation for 24 h, the cells were treated with dasatinib for 14 days. Colonies were fixed with 100% methanol and stained with 0.5% crystal violet solution [100]. Plates were scanned using G-Box (Synoptics, Cambridge, UK), and images were analyzed using ImageJ as described previously by Guzman et al. [101].

### 4.13. Statistics

Testing for statistical significance was performed by ANOVA followed by the Tukey’s post hoc test. When two means were compared, student’s *t*-test was used. When *p* < 0.05, the observed difference was considered statistically significant.

## 5. Conclusions

In conclusion, our results show for the first time that SIRT2 is an important regulator of the basic functions and genes of melanoma cells, including those involved in cell motility, proliferation and drug resistance. We provide new tools in the form of cell lines lacking *SIRT2* expression to study the role of this sirtuin in melanoma cells. Low *SIRT2* levels alter the expression of tyrosine kinase receptors and induce melanoma cell susceptibility to the multikinase inhibitor dasatinib. Thus, we explained one of the possible resistance mechanisms to this drug. As a consequence of this finding, to improve therapeutic potential of present regiments against melanoma, inhibition of sirtuin 2 activity should be considered.

## Figures and Tables

**Table 1:** Selected category of genes (based on a literature search) with changes in expression in WM853 cells with *SIRT2* downregulation as determined using RNA-seq.

Gene Symbol	Protein	Effect	Average Fold Change
Integrins			
*ITGA1*	integrin subunit alpha 1	↓	0.462
*ITGA3*	integrin subunit alpha 3	↑	2.173
*ITGA4*	integrin subunit alpha 4	↓	0.556
*ITGA5*	integrin subunit alpha 5	↑	1.588
*ITGA6*	integrin subunit alpha 6	↑	2.221
*ITGA9*	integrin subunit alpha 9	↑	2.498
*ITGA11*	integrin subunit alpha 11	↓	0.551
*ITGAE*	integrin subunit alpha E	↓	0.540
*ITGAL*	integrin subunit alpha L	↓	0.540
**Cadherins**			
*CDH2*	cadherin 2	↑	1.770
*CDH19*	cadherin 19	↑	4.203
**Selectins**			
*SELL*	Selectin L	↑	8.589
**Syndecans**			
*SDC1*	syndecan 1	↓	0.300
*SDC2*	syndecan 2	↑	4.304
**Immunoglobulin-like Cell Adhesion Molecules**			
*NCAM1*	neural cell adhesion molecule	↑	21.328
**Receptor tyrosine kinases**			
*EGFR*	epidermal growth factor receptor	↓	0.456
*IGF1R*	insulin like growth factor 1 receptor	↑	1.653
*IGFLR1*	IGF like family receptor 1	↓	0.647
*FGFR1*	fibroblast growth factor receptor 1	↑	4.052
*NGFR*	nerve growth factor receptor	↓	0.579
*EPHA2*	EPH receptor A2	↓	0.477
*EPHA3*	EPH receptor A3	↑	1.646
*EPHA5*	EPH receptor A3	↓	0.421
*EPHB3*	EPH receptor B3	↑	2.767
*AXL*	AXL receptor tyrosine kinase	↓	0.592
*ROR1*	receptor tyrosine kinase like orphan receptor 1	↑	2.547
**MAP/ERK pathway**			
*GRB14*	growth factor receptor bound protein 14	↑	3.678
*RASAL1*	RAS protein activator like 1	↑	7.833
*RASD2*	RASD family member 2	↑	3.716
*RASEF*	RAS and EF-hand domain containing	↑	2.321
*RASGEF1C*	RasGEF domain family member 1C	↓	0.195
*RASGRP1*	RAS guanyl releasing protein 1	↑	6.635
*RASGRP3*	RAS guanyl releasing protein 3	↓	0.417
*RASSF2*	Ras association domain family member 2	↑	2.186
*RASSF4*	Ras association domain family member 4	↓	0.594
*RASSF5*	Ras association domain family member 5	↑	2.972
*MAP2K3*	mitogen-activated protein kinase kinase 3	↓	0.593
*MAP2K6*	mitogen-activated protein kinase kinase 6	↑	2.309
*MAP3K4*	mitogen-activated protein kinase kinase kinase 4	↓	0.598
*MAP3K14*	mitogen-activated protein kinase kinase kinase 14	↓	0.585
*MAP3K21*	mitogen-activated protein kinase kinase kinase 21	↑	3.521
**Other protein kinases**			
*BTK*	tyrosine-protein kinase BTK	↓	0.342
*GAK*	cyclin G-assiciated kinase	↓	0.583
*LYN*	tyrosine protein kinase LYN	↓	0.275
**Cell cycle regulators**			
*CDK5R1*	cyclin dependent kinase 5 regulatory subunit 1	↓	0.653
*CDK6*	Cyclin-dependent kinase 6	↑	1.660
*CDKL2*	cyclin dependent kinase like 2	↑	2.001
*CDKN1A*	Cyclin-dependent kinase inhibitor 1A (P21WAF)	↓	0.615
*CDKN2B*	cyclin dependent kinase inhibitor 2B	↑	4.107
*CDKN2C*	cyclin dependent kinase inhibitor 2C	↓	0.546
*CDKN2D*	cyclin dependent kinase inhibitor 2D	↓	0.587

Average fold change: the difference in gene expression levels between WM853 SCW3 and WM853 SSW30 clones was counted as the mean fold change in the deseq, deseq2 and edgeR analyses.

**Table 2:** Selected category of genes (based on a literature search) with changes in expression in MDA-MB-435S cells with *SIRT2* downregulation as determined using RNA-seq.

Gene Symbol	Protein	Effect	Average Fold Change
**Integrins**			
*ITGA1*	integrin subunit alpha 1	↓	0.051
*ITGBL1*	integrin subunit beta like 1	↓	0.227
**Cadherins**			
*CDH12*	cadherin 12	↑	8.313
**Syndecans**			
*SDC2*	syndecan 2	↓	0.452
**Immunoglobulin-like Cell Adhesion Molecules**			
*NCAM1*	neural cell adhesion molecule	↓	0.572
*ICAM5*	intercellular adhesion molecule 5	↓	0.449
**Receptor tyrosine kinases**			
*EGFR*	epidermal growth factor receptor	↓	0.195
*FGFR1*	fibroblast growth factor receptor 1	↑	2.005
*EPHA2*	EPH receptor A2	↓	0.423
*EPHB1*	EPH receptor B1	↓	0.360
*AXL*	AXL receptor tyrosine kinase	↑	2.086
*DDR1*	Discoidin domain receptor family, member 1	↓	0.576
**MAP/ERK pathway**			
*GRB14*	growth factor receptor bound protein 14	↓	0.234
*RASL10B*	RAS like family 10 member B	↑	2.220
*MAP3K9*	mitogen-activated protein kinase kinase kinase 9	↓	0.638
*MAPK8IP1*	mitogen-activated protein kinase 8 interacting protein 1	↓	0.342
*MAPK8IP2*	mitogen-activated protein kinase 8 interacting protein 2	↓	0.587
**Cell cycle regulators**			
*CDK14*	Cyclin-dependent kinase 14	↓	0.207
*CDK18*	Cyclin-dependent kinase 18	↑	1.536
*CDK20*	Cyclin-dependent kinase 20	↓	0.073
*CDKN2D*	cyclin dependent kinase inhibitor 2D	↓	0.643

Average fold change: the difference in gene expression levels between MDA-MB-435S SCM1 and MDA-MB-435S SSM15 clones was counted as the mean fold change in the deseq, deseq2 and edgeR analyses.

## Supplementary Materials

The following are available online at https://www.mdpi.com/2072-6694/11/5/673/s1, Figure S1: Phenotypes of the SIRT2-deficient melanoma cell lines, Figure S2: Venn diagrams to compare the identified DEGs from the different expression analysis tools and designate the shared DEGs, Figure S3: Individual cytotoxic effects of dasatinib on melanoma cell lines, Figure S4: Computer-generated area lacking values to illustrate the in vitro scratch assay from Figure 3, Table S1: Gene ontology (biological process) term results from the PANTHER overrepresentation test for significantly differentially expressed (DE) genes regulated in both melanoma cells analyzed, Table S2: Expression of selected genes in A375 melanoma cells after treatment with the SIRT2 inhibitor thiomyristoyl, DataSet S1: A complete list of genes that changed expression in WM853 cell line after SIRT2 downregulation determined using RNA-seq, DataSet S2: A complete list of genes that changed expression in MDA-MB-435S cell line after SIRT2 downregulation determined using RNA-seq, DataSet S3: A complete list of gene ontology (biological process) term results from the PANTHER overrepresentation test for significantly differentially expressed (DE) genes regulated in WM853 SCW3/WM853 SSW30 and MDA-MB-435S SCM1/MDA-MB-435S SSM15 cells, Original scans of Figure 1, Figure 5 and Figure 6.
