# Supplementary material for: SIRT2 Contributes to the Resistance of Melanoma Cells to the Multikinase Inhibitor Dasatinib
Source: Cancers (Basel). 2019 May 14;11(5):673. doi: 10.3390/cancers11050673 (PMC6562913; doi:10.3390/cancers11050673)
Supplement: Supplementary file 1 [file cancers-11-00673-s001.zip › cancers-489669 - supplementary files/cancers-489669 - supplementary-final check.docx]

Supplementary Materials: SIRT2 Contributes to the Resistance of Melanoma Cells to the Multikinase Inhibitor Dasatinib

Iwona Karwaciak, Anna Sałkowska, Kaja Karaś, Marta Sobalska-Kwapis, Aurelia Walczak-Drzewiecka, Łukasz Pułaski, Dominik Strapagiel, Jarosław Dastych and Marcin Ratajewski

Supplementary Figures


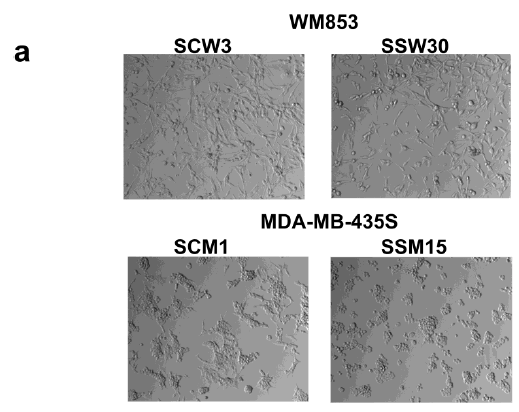

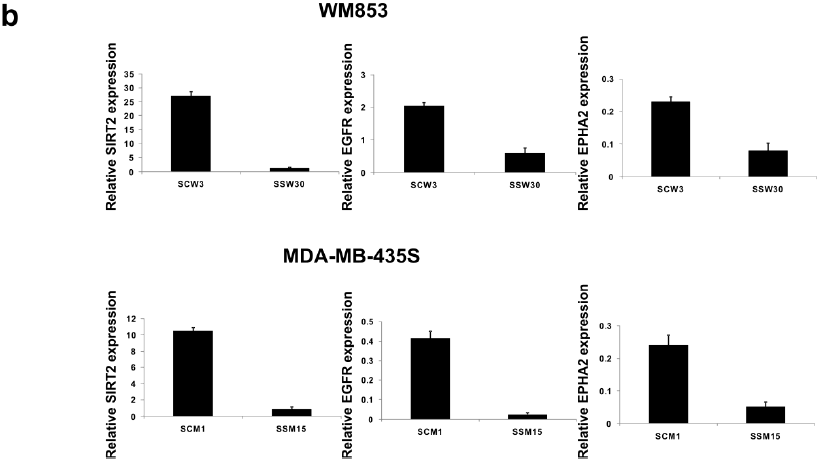


**Figure S1.** Phenotypes of the *SIRT2*-deficient melanoma cell lines. (**a**) Phenotypes of SCW3 and SSW30 clones of WM853 cells and SCM1 and SSM15 clones of MDA-MB-435S (photo from an optical microscope). 10 × 10 magnification. (**b**) *SIRT2*, *EGFR* and *EPHA2* expression in SCW3 and SSW30 clones of WM853 cells and SCM1 and SSM15 clones of MDA-MB-435S, as determined using quantitative PCR and normalized to the expression of the housekeeping gene *RPL13A*, mean ± SD, *n* = 3.


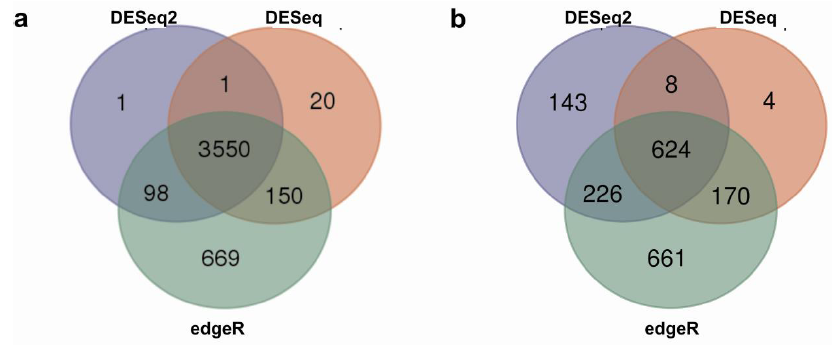


**Figure S2.** Venn diagrams to compare the identified DEGs from the different expression analysis tools and designate the shared DEGs. (**a**) WM853 SCW3 and WM853 SSW30 cells. (**b**) MDA-MB-435S SCM1 and MDA-MB-435S SSM15 cells.


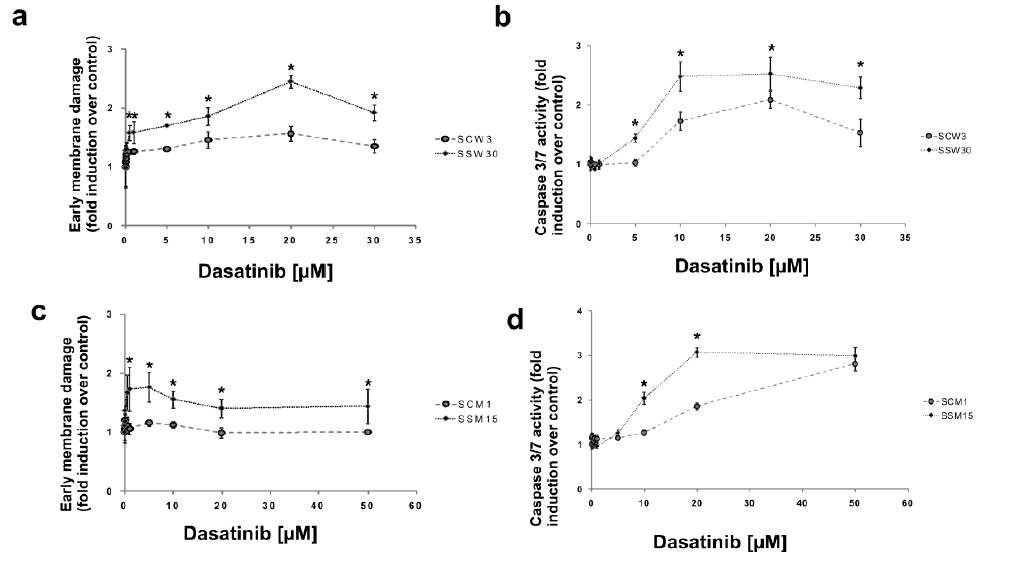


**Figure S3.** Individual cytotoxic effects of dasatinib on melanoma cell lines. (**a**) Effect of dasatinib on early membrane damage in SCW3 and SSW30 clones; mean ± SD, *n* = 6. * Indicates a statistically significant difference at *p* < 0.05. (**b**) Effect of dasatinib on apoptosis in SCW3 and SSW30 clones as measured by increased caspase 3/7 activity; mean ± SD, *n* = 6. * Indicates a statistically significant difference at *p* < 0.05. (**c**) Effect of dasatinib on early membrane damage in SCM1 and SSM15 clones; mean ± SD, *n* = 6. * Indicates a statistically significant difference at *p* < 0.05. (**d**) Effect of dasatinib on apoptosis in SCM1 and SSM15 clones as measured by increased caspase 3/7 activity; mean ± SD, *n* = 6. * Indicates a statistically significant difference at *p* < 0.05.


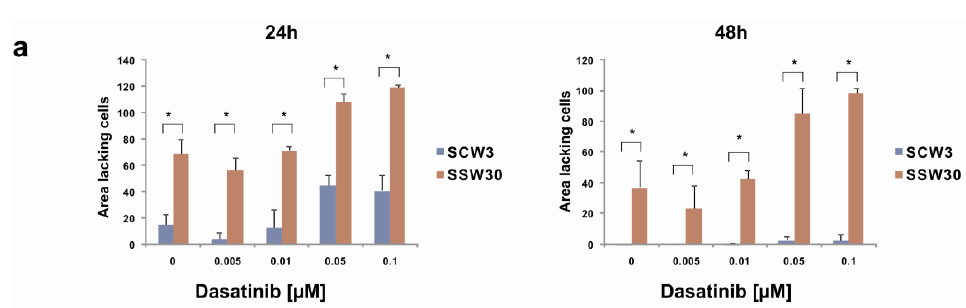

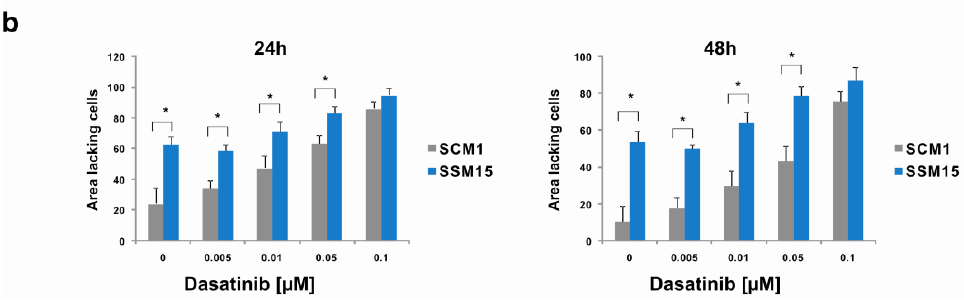


**Figure S4.** Computer-generated area lacking values to illustrate the in vitro scratch assay from Figure 3. (**a**) Results of the scratch assay of the WM853 SCW3 and WM853 SSW30 after 24 and 48 h. (**b**) Results of the scratch assay of the MDA-MB-435S SCM1 and MDA-MB-435S SSM15 after 24 and 48 h. The data represent the mean ± SD, (*n* = 4). * Indicates a statistically significant difference at *p* < 0.05.

Supplementary Tables

**Table S1.** Gene ontology (biological process) term results from the PANTHER overrepresentation test for significantly differentially expressed (DE) genes regulated in both melanoma cells analyzed. GOID: Gene Ontology term identifier; number of genes: the number of genes mapped to a specific GO term; *p*-value: the expected value calculated by Fisher’s exact test with false discovery rate multiple test correction (*p* < 0.05) for overrepresentation of selected DE genes in the GO category.

| **Cells** | | **WM853** (**Clones SCW3 and SSW30**) | | **MDA-MB-435S** (**Clones SCM1 and SSM15**) | |
| --- | --- | --- | --- | --- | --- |
| **GO ID** | **GO Biological Process Term** | **Number of Genes** | ***p*-Value** | **Number of Genes** | ***p*-Value** |
| GO:0007155 | cell adhesion | 189 | 4.61 × 10^-11^ | 74 | 6.77 × 10^-18^ |
| GO:0098609 | cell-cell adhesion | 92 | 9.56 × 10^-05^ | 35 | 7.67 × 10^-08^ |
| GO:0030155 | regulation of cell adhesion | 128 | 1.41 × 10^-06^ | 39 | 2.15 × 10^-06^ |
| GO:0031589 | cell-substrate adhesion | 39 | 6.31 × 10^-04^ | 17 | 3.60 × 10^-06^ |
| GO:0016477 | cell migration | 171 | 2.83 × 10^-07^ | 63 | 2.91 × 10^-12^ |
| GO:0048870 | cell motility | 181 | 2.66 × 10^-06^ | 66 | 1.25 × 10^-11^ |
| GO:2000145 | regulation of cell motility | 191 | 1.77 × 10^-13^ | 49 | 2.50 × 10^-07^ |
| GO:0030334 | regulation of cell migration | 187 | 6.67 × 10^-15^ | 46 | 6.02 × 10^-07^ |
| GO:0008283 | cell proliferation | 125 | 2.89 × 10^-05^ | 37 | 2.31 × 10^-05^ |
| GO:0042127 | regulation of cell proliferation | 315 | 1.96 × 10^-15^ | 63 | 5.06 × 10^-04^ |
| GO:0010941 | regulation of cell death | 296 | 1.10 × 10^-09^ | 66 | 4.79 × 10^-04^ |
| GO:0045595 | regulation of cell differentiation | 349 | 1.74 × 10^-18^ | 89 | 3.15 × 10^-10^ |
| GO:0030154 | cell differentiation | 640 | 2.01 × 10^-22^ | 139 | 1.89 × 10^-07^ |
| GO:0048583 | regulation of response to stimulus | 683 | 1.40 × 10^-15^ | 170 | 7.63 × 10^-11^ |
| GO:0009605 | response to external stimulus | 375 | 3.09 × 10^-15^ | 86 | 1.68 × 10^-06^ |
| GO:0010033 | response to organic substance | 517 | 2.73 × 10^-18^ | 113 | 2.13 × 10^-06^ |
| GO:0050896 | response to stimulus | 1209 | 2.13 × 10^-15^ | 261 | 6.74 × 10^-06^ |
| GO:0009719 | response to endogenous stimulus | 252 | 1.35 × 10^-08^ | 59 | 2.26 × 10^-04^ |

**Table S2.** Expression of selected genes in A375 melanoma cells after treatment with the SIRT2 inhibitor thiomyristoyl. Cells were treated with 50 μM thiomyristoyl for 48 h and then were subjected for RNA extraction. Expression of the cognate mRNA was determined using quantitative PCR and normalized to that of the housekeeping gene RPL13A, mean ± SD, *n* = 3. * Indicates a statistically significant difference at *p* < 0.05.

| **Gene** | | **Control** | | **Thiomyristoyl** |
| --- | --- | --- | --- | --- |
| *EGFR* | 1.40 ± 0.14 | | 0.84 ± 0.17 * | |
| *EPHA2* | 1.65 ± 0.13 | | 1.26 ± 0.07 * | |
| *ITGA1* | 21.8 ± 1.2 | | 3.6 ± 1.1 * | |
| *SDC2* | 11.7 ± 0.80 | | 5.1 ± 0.8 * | |
| *GAK* | 1.92 ± 0.22 | | 1.76 ± 0.29 | |
| *LYN* | 0.639 ± 0.049 | | 0.517 ± 0.080 | |
| *DDR1* | 1.22 ± 0.15 | | 0.62 ± 0.09 * | |
| *EPHB1* | 0.479 ± 0.048 | | 0.219 ± 0.032 * | |

DataSet S1–S3: please find at supplementary files

Original Scans of Figures 1, 5 and 6

Original scans of Figures 1

SIRT2

Size marker, MDA-MB-435S, SCM1, SSM15, size marker


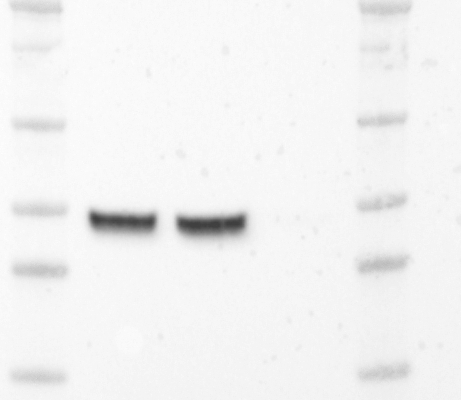


β-actin

size marker, MDA-MB-435S, SCM1, SSM15, size marker


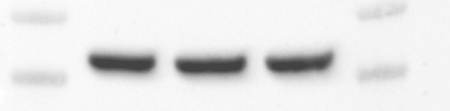


SIRT2

Size marker, WM853, SCW3, SSW30


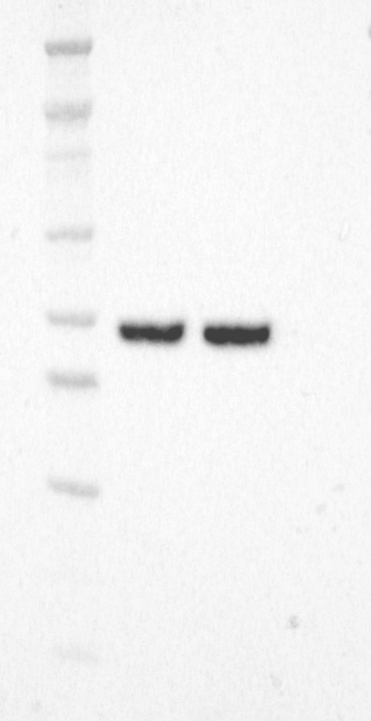


β-actin

Size marker, WM853, SCW3, SSW30


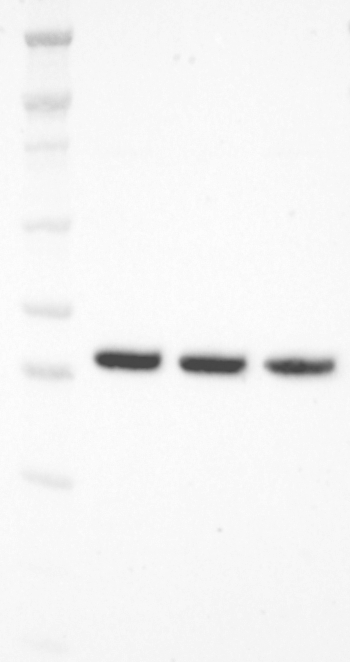


Original scans of Figures 5

SCM1 pEGFR

SCM1 ctrl; SCM1 EGF 50 ng/mL10 min; SCM1 EGF 50 ng/mL30 min; SCM1 EGF 200 ng/mL10 min


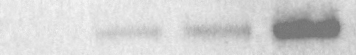


SSM15 pEGFR

SSM15 ctrl; SSM15 EGF 50 ng/mL10 min; SSM15 EGF 50 ng/mL30 min; SSM15 EGF 200 ng/mL10 min


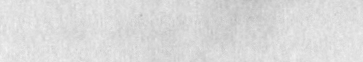


SCM1 EGFR

SCM1 ctrl; SCM1 EGF 50 ng/mL10 min; SCM1 EGF 50 ng/mL30 min; SCM1 EGF 200 ng/mL10 min


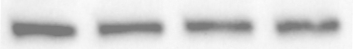


SSM15 EGFR

SSM15 ctrl; SSM15 EGF 50 ng/mL10 min; SSM15 EGF 50 ng/mL30 min; SSM15 EGF 200 ng/mL10 min


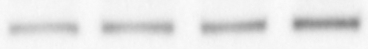


SCM1 β actin

SCM1 ctrl; SCM1 EGF 50 ng/mL10 min; SCM1 EGF 50 ng/mL30 min; SCM1 EGF 200 ng/mL10 min


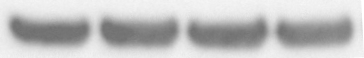


SSM15 β actin

SSM15 ctrl; SSM15 EGF 50 ng/mL10 min; SSM15 EGF 50 ng/mL30 min; SSM15 EGF 200 ng/mL10 min


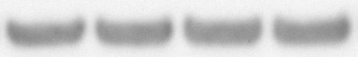


SCW3 pEGFR SCW3 ctrl; SCW3 EGF 50 ng/mL10 min; SCW3 EGF 50 ng/mL30 min; SCW3 EGF 200 ng/mL10 min


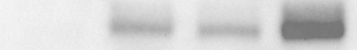


SSW30 pEGFR

SSW30 ctrl; SSW30 EGF 50 ng/mL10 min; SSW30 EGF 50 ng/mL30 min; SSW30 EGF 200 ng/mL10 min


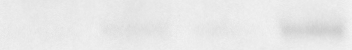


SCW3 EGFR

SCW3 ctrl; SCW3 EGF 50 ng/mL10 min; SCW3 EGF 50 ng/mL30 min; SCW3 EGF 200 ng/mL10 min


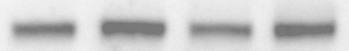


SSW30 EGFR

SSW30 ctrl; SSW30 EGF 50 ng/mL10 min; SSW30 EGF 50 ng/mL30 min; SSW30 EGF 200 ng/mL10 min


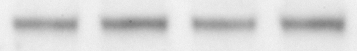


SCW3 β actin

SCW3 ctrl; SCW3 EGF 50 ng/mL10 min; SCW3 EGF 50 ng/mL30 min; SCW3 EGF 200 ng/mL10 min


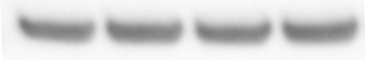


SSW30 β actin

SSW30 ctrl; SSW30 EGF 50 ng/mL10 min; SSW30 EGF 50 ng/mL30 min; SSW30 EGF 200 ng/mL10 min


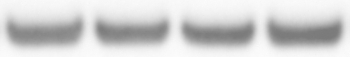


pEPHA2 (TYR588)

SCM1 ctrl; SCM1 ephrin-A1/Fc 500 ng/mL10 min; SSM15 ctrl; SSM15 ephrin-A1/Fc 500 ng/mL10 min size marker; SCM1 ctrl; SCM1 ephrin-A1/Fc 100 ng/mL30 min; SSM15 ctrl; SSM15 ephrin-A1/Fc 100 ng/mL30 min


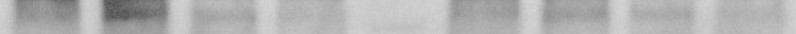


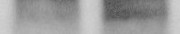

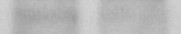


pEPHA2 cst6347

SCM1 ctrl; SCM1 ephrin-A1/Fc 500 ng/mL10 min; SSM15 ctrl; SSM15 ephrin-A1/Fc 500 ng/mL10 min size marker; SCM1 ctrl; SCM1 ephrin-A1/Fc 100 ng/mL30 min; SSM15 ctrl; SSM15 ephrin-A1/Fc 100 ng/mL30 min


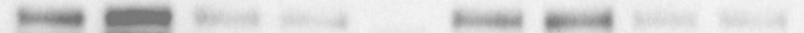


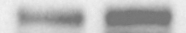

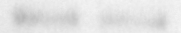


β actin ab8227

SCM1 ctrl; SCM1 ephrin-A1/Fc 500 ng/mL10 min; SSM15 ctrl; SSM15 ephrin-A1/Fc 500 ng/mL10 min size marker; SCM1 ctrl; SCM1 ephrin-A1/Fc 100 ng/mL30 min; SSM15 ctrl; SSM15 ephrin-A1/Fc 100 ng/mL30 min


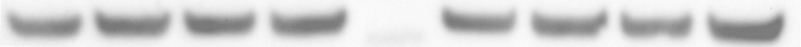


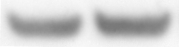

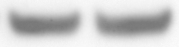


pEPHA2 cst12677

SCW3 ctrl; SCW3 ephrin-A1/Fc 100 ng/mL30 min; SCW3 ephrin-A1/Fc 500 ng/mL10 min; SSW30 ctrl; SSW30 ephrin-A1/Fc 100 ng/mL30 min; SSW30 ephrin-A1/Fc 500 ng/mL10 min


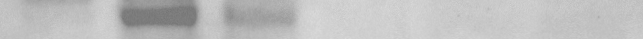


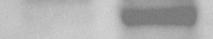

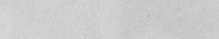


β actin ab8227

SCW3 ctrl; SCW3 ephrin-A1/Fc 100 ng/mL30 min; SCW3 ephrin-A1/Fc 500 ng/mL10 min; SSW30 ctrl; SSW30 ephrin-A1/Fc 100 ng/mL30 min; SSW30 ephrin-A1/Fc 500 ng/mL10 min


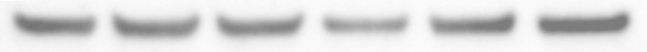


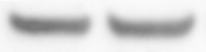

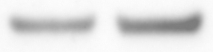


Original scans of Figures 6

MDA-MB-435S

pEPHA2 (Ser897) cst6347

SCM ctrl; SCM das 0,5 µM 1 h; SCM das 1 µM 1 h; SSM ctrl; SSM das 0,5 µM 1 h; SSM das 1 µM 1 h


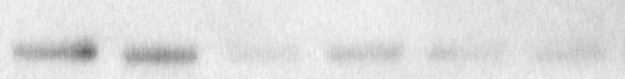


pEPHA2 (Tyr588) GTX32176

SCM ctrl; SCM das 0,5 µM 1 h; SCM das 1 µM 1 h; SSM ctrl; SSM das 0,5 µM 1 h; SSM das 1 µM 1 h


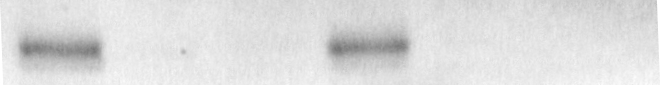


EPHA2 cst6997

SCM ctrl; SCM das 0,5 µM 1 h; SCM das 1 µM 1 h; SSM ctrl; SSM das 0,5 µM 1 h; SSM das 1 µM 1 h


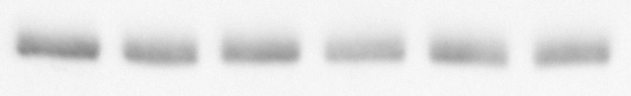


pEGFR cst3777

SCM ctrl; SCM das 0,5 µM 1 h; SCM das 1 µM 1 h; SSM ctrl; SSM das 0,5 µM 1 h; SSM das 1 µM 1 h


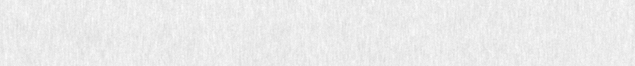


EGFR cst2232

SCM ctrl; SCM das 0,5 µM 1 h; SCM das 1 µM 1 h; SSM ctrl; SSM das 0,5 µM 1 h; SSM das 1 µM 1 h


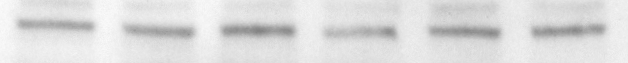


pFAK cst3281

SCM ctrl; SCM das 0,5 µM 1 h; SCM das 1 µM 1 h; SSM ctrl; SSM das 0,5 µM 1 h; SSM das 1 µM 1 h


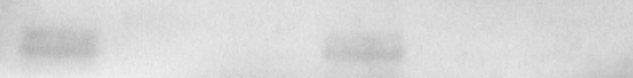


FAK cst3285

SCM ctrl; SCM das 0,5 µM 1 h; SCM das 1 µM 1 h; SSM ctrl; SSM das 0,5 µM 1 h; SSM das 1 µM 1 h


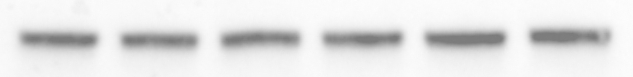


pSRC cst2101

SCM ctrl; SCM das 0,5 µM 1 h; SCM das 1 µM 1 h; SSM ctrl; SSM das 0,5 µM 1 h; SSM das 1 µM 1 h


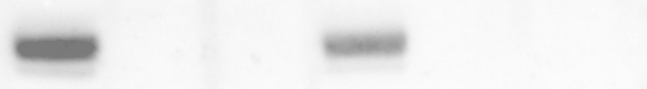


SRC cst2108

SCM ctrl; SCM das 0,5 µM 1 h; SCM das 1 µM 1 h; SSM ctrl; SSM das 0,5 µM 1 h; SSM das 1 µM 1 h


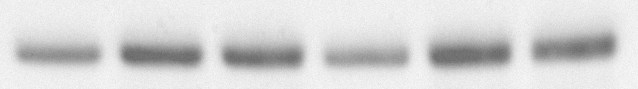


pERK sc7383

SCM ctrl; SCM das 0,5 µM 1 h; SCM das 1 µM 1 h; SSM ctrl; SSM das 0,5 µM 1 h; SSM das 1 µM 1 h


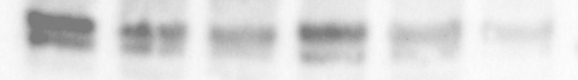


ERK sc514302

SCM ctrl; SCM das 0,5 µM 1 h; SCM das 1 µM 1 h; SSM ctrl; SSM das 0,5 µM 1 h; SSM das 1 µM 1 h


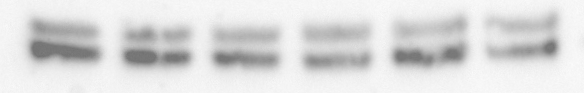


Β-actin ab8227

SCM ctrl; SCM das 0,5 µM 1 h; SCM das 1 µM 1 h; SSM ctrl; SSM das 0,5 µM 1 h; SSM das 1 µM 1 h


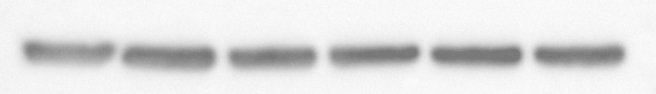


WM853

pEPHA2 (Ser897) cst6347

SCW ctrl; SCW das 0,5 µM 1 h; SCW das 1 µM 1 h; SSW ctrl; SSW das 0,5 µM 1 h; SSW das 1 µM 1 h


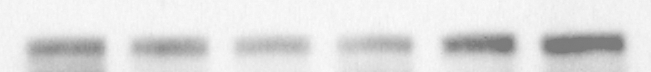


pEPHA2 (Tyr588) GTX32176

SCW ctrl; SCW das 0,5 µM 1 h; SCW das 1 µM 1 h; SSW ctrl; SSW das 0,5 µM 1 h; SSW das 1 µM 1 h


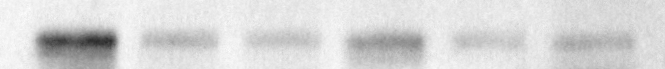


EPHA2 cst6997

SCW ctrl; SCW das 0,5 µM 1 h; SCW das 1 µM 1 h; SSW ctrl; SSW das 0,5 µM 1 h; SSW das 1 µM 1 h


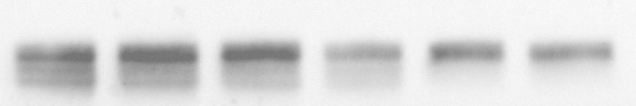


pEGFR cst3777

SCW ctrl; SCW das 0,5 µM 1 h; SCW das 1 µM 1 h; SSW ctrl; SSW das 0,5 µM 1 h; SSW das 1 µM 1 h


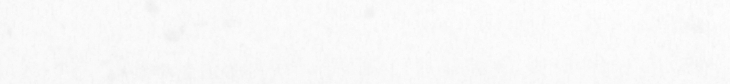


EGFR cst2232

SCW ctrl; SCW das 0,5 µM 1 h; SCW das 1 µM 1 h; SSW ctrl; SSW das 0,5 µM 1 h; SSW das 1 µM 1 h


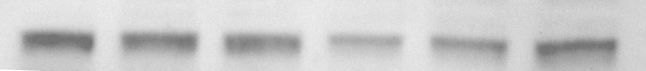


pFAK cst3281

SCW ctrl; SCW das 0,5 µM 1 h; SCW das 1 µM 1 h; SSW ctrl; SSW das 0,5 µM 1 h; SSW das 1 µM 1 h


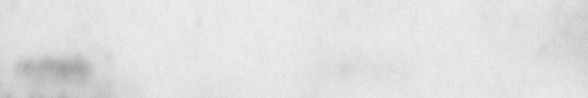


FAK cst3285


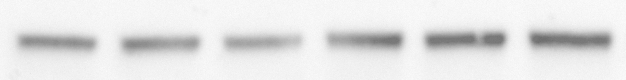


pSRC cst2101

SCW ctrl; SCW das 0,5 µM 1 h; SCW das 1 µM 1 h; SSW ctrl; SSW das 0,5 µM 1 h; SSW das 1 µM 1 h


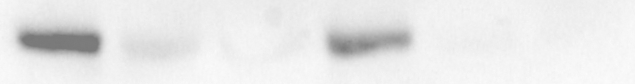


SRC cst2108

SCW ctrl; SCW das 0,5 µM 1 h; SCW das 1 µM 1 h; SSW ctrl; SSW das 0,5 µM 1 h; SSW das 1 µM 1 h


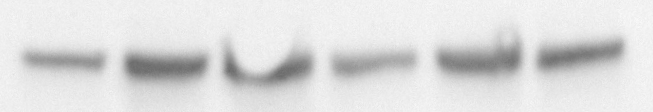


pERK sc7383

SCW ctrl; SCW das 0,5 µM 1 h; SCW das 1 µM 1 h; SSW ctrl; SSW das 0,5 µM 1 h; SSW das 1 µM 1 h


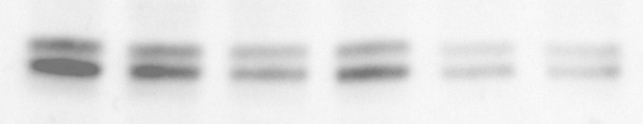


ERK sc514302

SCW ctrl; SCW das 0,5 µM 1 h; SCW das 1 µM 1 h; SSW ctrl; SSW das 0,5 µM 1 h; SSW das 1 µM 1 h


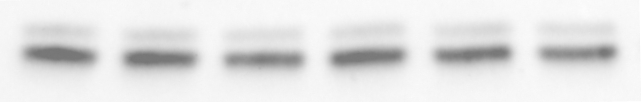


Β-actin ab8227

SCW ctrl; SCW das 0,5 µM 1 h; SCW das 1 µM 1 h; SSW ctrl; SSW das 0,5 µM 1 h; SSW das 1 µM 1 h


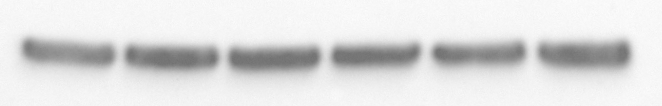


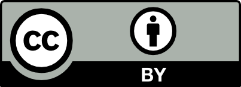
© 2019 by the authors. Licensee MDPI, Basel, Switzerland. This article is an open access article distributed under the terms and conditions of the Creative Commons Attribution (CC BY) license (http://creativecommons.org/licenses/by/4.0/).
